# Estimating the Effect of Aerobic Exercise Training on Novel Lipid Biomarkers: A Systematic Review and Multivariate Meta-Analysis of Randomized Controlled Trials

**DOI:** 10.1007/s40279-023-01817-0

**Published:** 2023-03-02

**Authors:** Gina Wood, Emily Taylor, Vanessa Ng, Anna Murrell, Aditya Patil, Tom van der Touw, Mitch Wolden, Nick Andronicos, Neil A. Smart

**Affiliations:** 1 School of Science and Technology in the Faculty of Science, Agriculture, Business and Law, University of New England, Armidale, NSW 2351 Australia; 2 School of Allied Health, Curtin University, Bentley, WA 6102 Australia; 3 School of Rural Medicine in the Faculty of Medicine and Health, University of New England, Armidale, NSW 2351 Australia; 4 Physical Therapy Program, University of Jamestown, Fargo, ND 58104 USA

## Abstract

**Background:**

Aerobic exercise training (AET) prescribed as lipid management treatment positively affects the standard lipid profile and reduces cardiovascular disease (CVD) risk. Apolipoproteins, lipid and apolipoprotein ratios, and lipoprotein sub-fractions may more effectively predict CVD risk than the standard lipid profile but an AET response in these biomarkers has not been established.

**Objectives:**

We conducted a quantitative systematic review of randomised controlled trials (RCTs) to (1) determine the effects of AET on lipoprotein sub-fractions, apolipoproteins and relevant ratios; and (2) identify study or intervention covariates associated with change in these biomarkers.

**Methods:**

We searched PubMed, EMBASE, all Web of Science and EBSCO health and medical online databases from inception to 31 December 2021. We included published RCTs of adult humans with ≥ 10 per group of participants; an AET intervention duration ≥ 12 weeks of at least moderate intensity (> 40% maximum oxygen consumption); and reporting pre/post measurements. Non-sedentary subjects, or those with chronic disease other than Metabolic Syndrome factors, or pregnant/lactating, as well as trials testing diet/medications, or resistance/isometric/unconventional training interventions, were excluded.

**Results:**

Fifty-seven RCTs totalling 3194 participants were analysed. Multivariate meta-analysis showed AET significantly raised antiatherogenic apolipoproteins and lipoprotein sub-fractions (mmol/L mean difference (MD) 0.047 (95% confidence interval (CI) 0.011, 0.082), *P* = .01); lowered atherogenic apoliproteins and lipoprotein sub-fractions (mmol/L MD − 0.08 (95% CI − 0.161, 0.0003), *P* = .05); and improved atherogenic lipid ratios (MD − 0.201 (95% CI − 0.291, − 0.111), *P* < .0001). Multivariate meta-regression showed intervention variables contributed to change in lipid, sub-fraction, and apoliprotein ratios.

**Conclusion:**

Aerobic exercise training positively impacts atherogenic lipid and apolipoprotein ratios, alipoproteins, and lipoprotein sub-fractions; and antiatherogenic apolipoproteins and lipoprotein sub-fractions. Cardiovascular disease risk predicted by these biomarkers may be lowered when AET is prescribed as treatment or prevention.

**PROSPERO ID:**

CRD42020151925.

**Supplementary Information:**

The online version contains supplementary material available at 10.1007/s40279-023-01817-0.

## Key Points

**Table:** 

Aerobic exercise training (AET) lowers atherogenic apolipoprotein and lipoprotein sub-fractions and lipid ratios, and raises antiatherogenic apolipoproteins and lipoprotein sub-fractions, in sedentary adults.
AET volume (session minutes, sessions per week, aerobic training intensity, and intervention duration) is associated with positive change in atherogenic lipid, sub-fraction and apolipoprotein ratios.
Reporting of apolipoprotein, lipid and sub-fraction ratios is less common than standard lipid outcomes. Future AET trials should report these parameters as cardiovascular disease risk biomarkers.

## Introduction

The standard lipid profile (SLP) biomarkers used to evaluate cardiovascular (CVD) risk comprise total cholesterol (TC), triglycerides (TRG), high-density lipoprotein cholesterol (HDL-C) and low-density lipoprotein cholesterol (LDL-C) [1]. Dyslipidaemia, a lipid profile characterised by abnormally elevated or lowered lipids, is an important Metabolic Syndrome (MetS) risk factor of CVD [2, 3]. A recent 17-year follow-up study of females concluded the TC/HDL-C ratio was a potent predictor of CVD events [4]. A systematic review collating data from several large observational studies found TC/HDL-C and LDL-C/HDL-C ratios better predicted CVD risk than the SLP biomarkers [5].

Apolipoproteins (Apo) A1 and A2 are the largest protein constituent of HDL [6]. The Apo B100 contains an LDL-receptor responsible for the uptake of LDL, and serves to assemble and secrete VLDL [7]. Raised levels of Apo A1 and A2 are considered to be antiatherogenic, while increased levels of Apo B100 and VLDL are atherogenic [8]. Apolipoproteins and the Apo B100/Apo A1 ratio have been investigated as biomarkers more senstive to identifying CVD risk than TC, TRG and LDL-C [9–11]. Systematic reviews have examined the risk prediction power of Apo A1, A2 and B100 for cardiovascular risk and found Apo B100 and the Apo B100/Apo A1 ratio improved prediction [12–14]. Lowered levels of lipoprotein sub-fractions HDL2 and HDL3 are considered to increase CVD risk, although HDL3 may be less protective in the presence of MetS [15]. Sub-fractions of HDL-C may be more relevant in identifying CVD risk than HDL-C [11].

Lack of aerobic physical activity increases CVD risk [16]. Aerobic exercise training (AET) positively affects dyslipidaemia and MetS [17–20] and lowers CVD risk [21, 22]. Trials reporting the effects of exercise on apolipoproteins and lipid ratios suggest that exercise training exerts a positive effect on these biomarkers [23–25]; however, to the best of our knowledge, a comprehensive quantitative review investigating the effects of AET on apolipoproteins, lipoprotein sub-fractions, associated ratios and lipid ratios in adults free of chronic disease other than MetS factors has yet to be conducted. The number of pooled exercise trial analyses may be few because of the under-reporting of these biomarkers, or reporting in differing units of measurement, or disparity of health status in the investigated cohorts. A meta-analytical technique appropriate for missing or multiple correlated and non-independent outcomes, such as lipid ratios, lipoprotein sub-fractions and apolipoproteins, is multivariate meta-analysis of joined outcomes [26, 27]. We hypothesise that in a cohort of similar health status, AET will positively affect these biomarkers and should lead to a reduction in CVD risk.

We thus aimed to conduct a multivariate meta-analysis of RCTs comparing the effects of AET achieving a minimum aerobic intensity (> 40% maximum oxygen consumption (VO_2MAX_)), against no exercise on apolipoproteins, lipoprotein sub-fractions, associated ratios and lipid ratios in adults of like health status. Further, using multivariate meta-regression, we wanted to investigate whether a priori covariates were associated with change in outcome measures.

## Methods

This systematic review with multivariate meta-analysis and meta-regression was designed by GW and NS and registered in the International Prospective Register of Systematic Reviews (PROSPERO) [28], CRD42020151925. The results are presented according to the Preferred Reporting Items for Systematic Reviews and Meta-Analyses (PRISMA) statement [29].

### Data Sources

Potential studies were identified by systematic online searches of PubMed, EMBASE, all Web of Science and EBSCO health and medical databases from inception to 31 December 2021, for RCTs published in English language journals. Searches included a mix of Medical Subject Headings (MeSH) and free text terms (see Online Supplementary Material (OSM) Table S1 for search terms, exclusions and example search strategy). Other systematic reviews and reference lists of papers were hand searched for additional RCTs.

### Study Eligibility

Studies were eligible for inclusion if the study design was an RCT comparing an AET intervention against a non-exercising control group.

### Study Selection

GW, ET, AP and VN conducted online database searches and reviewed search results on the basis of title and abstract independently, using Microsoft Excel (Version 16.31 2019). GW, ET, AP and VN assessed and reviewed the full PDF texts of potentially eligible RCTs independently. NS was consulted to resolve discussion regarding the final list of RCTs for inclusion.

#### Participants

Studies of adult participants described as sedentary and capable of physical activity prior to the intervention and with no chronic disease, other than MetS factors (body mass index (BMI) ≥ 30 kg/m^2^; hypertensive blood pressure > 130/85 mm Hg; TRG ≥ 10.7 mmol/L; HDL-C < 1 mmol/L (men) or HDL-C < 1.3 mmol/L (women); fasting blood sugar > 5.5 mmol/L, diabetes mellitus type 1 or 2, or medication prescribed to manage these factors) [30–32] were included. Studies of intervention and control group population sample sizes (N) < 10 were excluded to reduce the likelihood of small study effects, for example over-estimation of effect size [33].

#### Intervention

An AET intervention ≥ 12 weeks was considered the minimum time to affect lipid profiles [34, 35]. Studies of either prescribed steady-state or interval AET that employed at least a moderate intensity effort (> 40% VO_2MAX_) were included, since this intensity is the minimum recommended for sedentary individuals [36, 37]. No restrictions were placed on AET session time or type. Other protocol inclusion and exclusion criteria are listed in Table S2 (OSM).

#### Comparator

An AET intervention was required to be compared to a non-exercising control group.

#### Outcomes

Pre- and post-intervention measurements in mass (mg/dL) or molar (mmol/L) units of measurement of lipoprotein sub-fractions, apolipoproteins, or associated ratios and lipid ratios, for each of intervention and non-exercising control groups, were required to be reported. Lipid sub-fractions measurements given in mg/dL were multiplied by 0.02586 to convert to mmol/L [38]. All Apo measurements, whether reported as mass or molar, remained unconverted. Lead authors of included studies were contacted via electronic correspondence for missing values of outcomes. Any outcome data presented graphically were converted to numerical values using WebPlotDigitzer (Version 4.2, 2019).

### Data Extraction

Pre-established data extraction sheets designed by GW, using Microsoft Excel (Version 16.31 2019), were populated with extracted data. Included RCTs were randomly divided between three teams (ET and VN; AP and TvdT; and AM and GW). Each team member independently extracted data from the RCTs and reviewed the other team member’s data extraction for accuracy. NS and NA resolved disagreements. The following data were extracted: (1) author(s), year of publication and study design; (2) demographic and clinical characteristics; (3) AET intervention and non-exercising control protocols; (4) intervention and control group intervention measurements for any Apo or lipoprotein sub-fractions, and lipid ratios, lipoprotein ratios, or Apo ratios; and (5) main findings. Summary data (mean (M) or mean difference (MD), standard deviation (SD) or standard error (SE), or either of SD or SE of the MD) were extracted from pre–post intervention and control group trials that used either within-group or between-group contrasts to define *P* values with 95% confidence intervals (CIs).

### Study Quality

Study quality was determined using the validated Tool for the Assessment of Study Quality and Reporting in Exercise (TESTEX) [39], a 15-point scale specific to exercise training studies (see Table S3 (OSM) for assessment criteria). A score ≥ 10 is considered good study quality and reporting [40]. Within-study risk-of-bias was evaluated against seven criteria (see Table S4 (OSM)) and a score of low, medium or high was awarded. Included RCTs were randomly distributed between each team, cross-checked for study quality data extraction accuracy, and reviewed by NS and NA. A study quality sub-analysis of RCTs grouped according to a TESTEX score ≥ 10 and a within-study risk evaluation of low-to-medium was conducted.

### Data Synthesis

Statistical analyses were performed using Comprehensive Meta-Analysis (CMA) 3.0 (Biostat, Inc., Englewood, NJ, USA). To allow for multiple missing and/or correlated outcomes [26, 27], we calculated point estimates (effect size) and 95% CIs using a continuous multivariate random effects model [41] with the effects measures of raw MD and SD. We set statistical significance (*P* value) at 5% for the effect size. This model assumes normal distribution of raw data. Outcomes were joined according to atherogenic potential, change of effect size direction, and unit of measurement (mmol/L or mg/dL). Outcomes that could not be joined were analysed using a univariate model with effects measures and significance as described for the above multivariate model. Reported effects measures for each of the intervention and control groups, whether analysis-by-protocol or intention-to-treat, were pooled when at least three outcomes per group were provided. If necessary and where possible, missing effects measures data were calculated as follows: the MD was calculated by subtracting *M*_pre-treatment_ from *M*_post-treatment_. The standard deviation of the MD was calculated thus: SD = square root [(SD_pre-treatment_)^2^ + (SD_post-treatment_)^2^ − (2*r* × SD_pre-treatment_ × SD_post-treatment_)], assuming a correlation coefficient *r* = 0.5, considered a conservative estimate [42]. GW and NS independently entered data in CMA, and reviewed each other’s files for accuracy.

#### Meta-Analysis and Sub-Analyses

Comprehensive Meta-Analysis permits joining non-independent or correlated outcomes and calculating a pooled mean or selecting the largest mean amongst pooled studies with various missing outcomes. Although the former may under-estimate effect and significance, it aids in avoiding type 1 errors and increases the potential accuracy of estimated effect sizes and CIs, and hence was selected as the appropriate method. A continuous random effects multivariate meta-analysis was conducted in CMA as follows: outcomes were joined, using the mean of multiple per-study non-independent and potentially correlated outcomes, to assess the impact of AET. In each continuous random effects multivariate meta-analysis of the outcomes, RCTs were sorted chronologically according to year of publication and hence analysed cumulatively (i.e. over time). For outcomes unable to be joined (either because of effect size direction or unit of measurement), RCTs were sorted chronologically according to year of publication and analysed cumulatively using a univariate, rather than multivariate, continuous random effects meta-analysis.

Sub-analyses were conducted in CMA for study quality using TESTEX scores (RCTs with a score ≥ 10) and within-study bias analysis (low to medium). Data were entered by GW and reviewed by NS for accuracy. A leave-one-out (*K* − 1, where *K* = total number of pooled RCTs, and each RCT is excluded once) sensitivity analysis was also performed to evaluate the influence of each RCT on the effect size of pooled data [43].

#### Small-Study Effects

Comprehensive meta-analysis was used to examine small-study effects and determine the likelihood of missing studies. Each of Rosenthal’s failsafe *N*, Orwin’s failsafe *N*, Duval and Tweedie’s trim-and-fill, Egger’s regression test, Begg and Mazumdar’s rank correlation test, and precision and standard error funnel plots, were used to test for possible small-study effects. Data were entered into CMA by GW and NS and independently cross-checked. MW reviewed the analyses.

#### Heterogeneity

Heterogeneity was quantified using the *Q* statistic, and the corresponding *P* value, *τ*^2^, *τ*, and *I*^2^ [41]. The *Q* statistic, and the corresponding *P* value, compared the differences among the calculated effect sizes; *τ*^2^ measured absolute between-study heterogeneity and the estimated SD (*τ*) [41]. The relative measure of heterogeneity *I*^2^ ranges from 0% (complete homogeneity) to 100% (complete heterogeneity) [44]. MW reviewed the analyses conducted in CMA.

#### Meta-Regression

Meta-regression was conducted in CMA without adjustment for *P* values using a random effects restricted maximum likelihood model with a Hartung–Knapp adjustment to detect whether any a priori study or intervention covariates might explain a change in statistically significant outcomes. A priori AET intervention covariates (intensity, frequency, session duration and intervention duration) that have been shown to influence lipid outcomes were analysed [19, 45, 46]. A priori study covariates were analysed for the potential to affect results by: (1) improved laboratory testing employed in recent RCTs (trial publication year); (2) under-powering of trials (total sample size); (3) correlation of similar outcomes (number of outcomes extracted per trial); and (4) quality of trials (study quality TESTEX score). Data were entered in CMA by GW and validated by NS and MW.

## Results

**Fig. 1:**
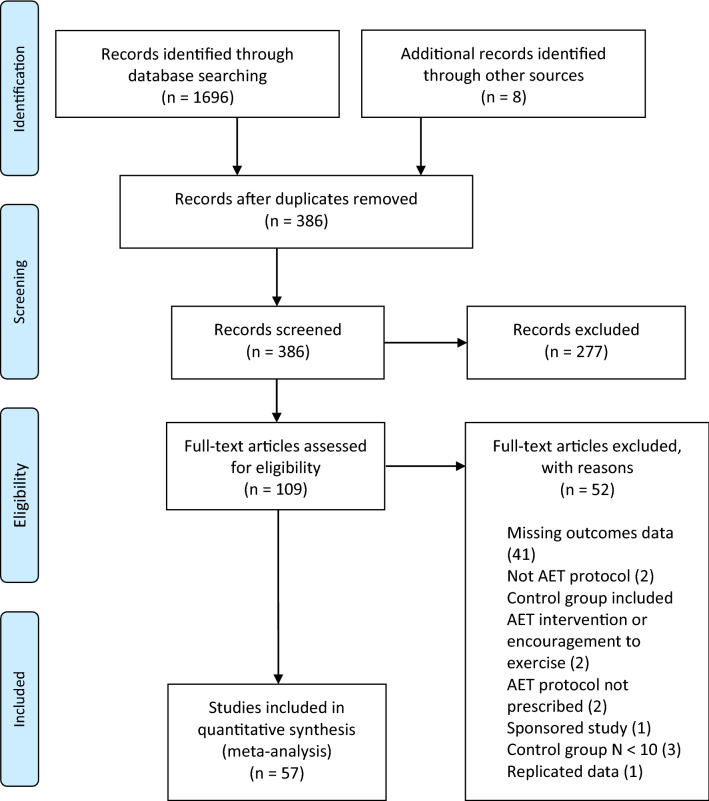
PRISMA (Preferred Reporting Items for Systematic Reviews and Meta-Analyses) flow diagram

The search and inclusion process is presented in Fig. 1 in a PRISMA flow diagram [29].

Combined searches resulted in a total of 1704 potential papers. After removal of duplicates and exclusion of articles based on abstract and title, 109 full-text articles remained for screening against inclusion and exclusion criteria. Screening resulted in the inclusion of 57 RCTs for data extraction, pooling and analysis, published between 1979 and 2020. We contacted three lead authors of whomh one lead author responded and provided data as requested. Two papers presented data graphically which was converted using WebPlotDigitzer (Version 4.2, 2019).

### Participant, and Intervention Characteristics

Details are provided in Table 1. Total participants numbered 3194 (exercise: 1721; control: 1473). Of these, 963 participants were female, 780 were male; the remaining 1451 participants were not classified. Participants under 35 years of age numbered 136, between 35–55 years of age there were 2060 participants, and 998 participants were aged over 55 years. All participants were sedentary before trial commencement and control groups were instructed to continue existing sedentary habits. Aerobic exercise intensity ranged from 40–80% VO_2MAX_. Intervention duration ranged from 12–52 weeks. Sessions per week ranged from 1.8 to 5.2, and minutes per session ranged from 15 to 210.

**Table 1:** Participant and intervention characteristics

Trial name	Total *N*	Age (range in years)	Sex	Number of extracted outcomes	Study quality score (/15)	Intensity VO_2MAX_ %	Intervention duration (weeks)	Sessions per week	Minutes per session
Aldred et al. [47]	22	35–55	F	2	10	59	12	4.6	29
Baker et al. [48]	34	> 55	M	1	9	72	20	3	48
Bell et al. MICT [49]	85	35–55	Mx	1	11	63	24	2.8	29
Boardley et al. [50]	68	> 55	Mx	1	9	65	16	3	35
Choi et al. [51]	75	35–55	F	2	10	50	12	5	60
Connolly et al. [52]	24	35–55	F	1	12	60	12	2.9	15
Costa et al. [53]	40	35–55	F	1	10	60	12	2	30
Finucane et al. [54]	87	> 55	Mx	1	12	60	12	3	60
Furukawa et al. [55]	45	35–55	F	2	12	50	12	2.5	30
Gahreman et al. [56]	24	< 35	M	1	13	75	12	3	20
Gordon et al. [57]	154	> 55	Mx	1	10	40	24	5	60
Grandjean et al. [58]	37	35–55	F	1	11	70	24	3	40
Hagan et al. a [59]	24	< 35	F	2	10	59	12	5	30
Hagan et al. b [59]	24	< 35	M	2	10	47	12	5	30
Hespel et al. [60]	27	35–55	M	4	9	80	16	3	40
Hinkleman & Nieman [61]	36	35–55	F	1	12	62	15	5	45
Huttunen et al. [62]	90	35–55	M	2	11	50	16	3.5	30
Kiens et al. [63]	37	35–55	M	1	8	80	12	2.6	45
Knoepfli-Lenzin et al. [64]	32	35–55	M	1	8	67	12	2.5	58
Korshøj et al. [65]	116	35–55	Mx	1	9	60	16	2	30
Krustrup et al. MICT [66]	31	35–55	F	1	10	70	16	1.8	52
Kukkonen-Harjula et al. [67]	108	35–55	Mx	2	12	70	15	3.8	45
Laaksonen et al. [68]	42	< 35	M	2	11	70	12	4	40
Lehmann et al. [69]	29	35–55	Mx	2	8	50	12	4	38
LeMura et al. [70]	22	< 35	F	1	9	59	16	3	30
Ligtenberg et al. [71]	51	> 55	Mx	3	11	70	26	3	50
Lindheim et al. [72]	45	35–55	F	4	9	52	26	3	30
Martins et al. [73]	63	> 55	Mx	1	6	60	16	3	45
Mohanka et al. [74]	173	> 55	F	2	12	57	52	3	45
Motoyama et al. [75]	30	> 55	Mx	1	12	50	39	5.2	30
Niederseer et al. [76]	34	> 55	Mx	2	10	55	12	2.4	210
Nieman et al. [77]	30	> 55	F	1	13	55	12	5	38
Nieman et al. [78]	43	35–55	F	1	13	65	12	4.8	45
Paolillo et al. [79]	20	35–55	F	2	12	79	52	2	45
Ready et al. [80]	25	> 55	F	3	8	54	26	4.9	54
Ring-Dimitriou et al. [81]	30	35–55	Mx	2	9	75	39	1	80
Rosenkilde et al. [82]	24	35–55	M	3	11	75	12	3	60
Rossi et al. [83]	33	> 55	F	1	7	70	16	2	52
Ruangthai and Phoemsapthawee [84]	25	> 55	Mx	2	11	48	24	3	40
Shearman et al. [85]	37	35–55	M	3	10	44	12	4.3	34
Sigal et al. [86]	123	35–55	Mx	2	15	75	22	2.4	45
Slentz et al. hvVICT [16]	84	35–55	Mx	1	10	73	26	3.6	58
Slentz et al. lvMICT [16]	72	35–55	Mx	1	10	48	26	3.5	58
Slentz et al. lvVICT [16]	83	35–55	Mx	1	10	73	26	2.9	43
Stefanick et al. a [87]	88	> 55	F	3	12	50	52	3	53
Stefanick et al. b [87]	93	35–55	M	3	12	50	52	3	53
Stensel et al. [88]	65	35–55	M	3	11	60	52	7	28
Sunami et al. [89]	40	> 55	Mx	3	10	50	22	3	60
Suter et al. [90]	61	35–55	M	1	9	77	16	3	30
Suter and Marti [91]	32	35–55	F	5	9	80	16	3	45
Tully et al. (=) [92]	52	35–55	Mx	1	13	53	12	4.2	26
Tully et al. (<) [92]	54	35–55	Mx	1	13	53	12	4.2	29
Verissimo et al. [93]	63	> 55	Mx	7	8	55	35	3	50
von Thiele Schwarz et al. [94]	118	35–55	F	1	8	49	52	3	60
Wirth et al. [95]	21	35–55	M	1	8	60	17	3	60
Wood et al. [96]	81	35–55	M	6	9	80	12	3	25
Wood et al. [97]	88	35–55	M	1	9	80	52	4	45
Total	3194		Median 1		10	60	16	3	45

*a* data reported for females, *b* data reported for males, *F* female, *hvVICT* high-volume vigorous-intensity continuous training, *lvMICT* low-volume moderate-intensity continuous training, *lvVICT* low-volume vigorous-intensity continuous training, *N* sample size, *M* male, *Mx* trial included both females and males, *VO*_*2MAX*_ maximum oxygen consumption, (=) exercise protocol equivalent to recommended exercise volumes, (<) exercise protocol less than recommended exercise volumes

Intervention protocols included weight-bearing activities such as running or walking on treadmills or outdoors, circuit training with no or minimal resistance components, and non-weight-bearing activities such as swimming, cycling and ergocycle. Trials included supervised and unsupervised training sessions. Effort was either unchanged or progressive in response to training adaptations. Measures of effort were clinically or self monitored and reported via training logs or electronic devices. These protocol attributes are further detailed in the Study Quality TESTEX and within-study risk-of-bias assessements.

### Comparative Outcomes

The ratio outcomes extracted from included RCTs were TC/HDL-C, LDL-C/HDL-C, HDL-C/TC, HDL-C/LDL-C, Apo B100/A1, and Apo A1/Apo B100. Sub-fractions extracted (mmol/L and mg/dL) were VLDL, HDL2 and HDL3. Apolipoproteins extracted (mmol/L and mg/dL) were Apo A1, Apo A2 and Apo B100.

Outcomes were joined according to antiatherogenicity, atherogenicity, effect size direction and reporting measurement. The TC/HDL-C, LDL-C/HDL-C and Apo B100/A1 ratios were joined (negative effect size direction) and analysed. The Apo A1/Apo B100, HDL-C/TC and HDL-C/LDL-C ratios were joined (positive effect size direction) and analysed. Apolipoprotein A1 and A2 mmol/L were joined with HDL2 and HDL3 mmol/L (antiatherogenic) and analysed. Apolipoprotein B100 mmol/L was joined with VLDL mmol/L (atherogenic) and analysed. Apolipoprotein A1 and A2 reported as mg/dL were joined (antiatherogenic) and analysed. Apolipoprotein B100 reported as mg/dL (atherogenic) was analysed separately. Summary statistics of the models are shown in Table 2.

**Table 2:** Multivariate and univariate random effects meta-analysis summary statistics per lipid outcome

Multivariate analysis model	Random effects, mean difference, 95% CI				Population *N*		
Mean of combined outcomes (apo, sub-fraction, ratio)	Point estimate	Lower limit	Upper lLimit	*P* value	Exercise	Control	Total
Apo A1 + A2 + HDL2 + HDL3 mmol/L	0.047	0.011	0.082	**0.010**	260	235	495
Apo A1 + A2 mg/dL	2.297	0.441	4.153	**0.015**	403	370	773
Apo B100 + VLDL mmol/L	− 0.053	− 0.114	0.008	0.087	535	360	895
TC/HDL-C + LDL-C/HDL-C + Apo B100/Apo A1	− 0.201	− 0.291	− 0.111	**0.000**	974	934	1908
HDL-C/TC + HDL-C/LDL-C + Apo A1/Apo B100	0.022	− 0.002	0.046	0.077	121	97	218

Univariate analysis model	Random effects, mean difference, 95% CI				Population *N*		
Single outcome	Point estimate	Lower limit	Upper limit	*P* value	Exercise	Control	Total
Apo B100 mg/dL	− 0.953	− 2.616	0.710	0.261	369	335	704

*Apo* apolipoprotein, *CI* confidence interval, *HDL/HDL-C* high-density lipoprotein(-cholesterol), *mg/dL* milligrams per decilitre, *mmol/L* millimoles per litre, *N* sample size per group and total, *TC* total cholesterol, *VLDL/LDL-C* (very) low-density lipoprotein(-cholesterol)

**Fig. 2:**
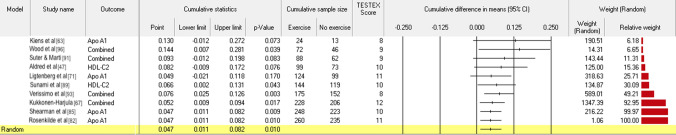
Antiatherogenic apolipoproteins (Apo) and sub-fractions (joined Apo A1 + A2 + HDL2 + HDL3 mmol/L)

**Fig. 3:**
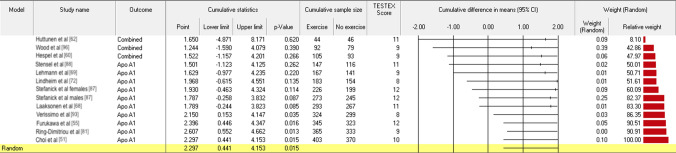
Antiatherogenic apolipoproteins (Apo) (joined Apo A1 + Apo A2 mg/dL)

**Fig. 4:**
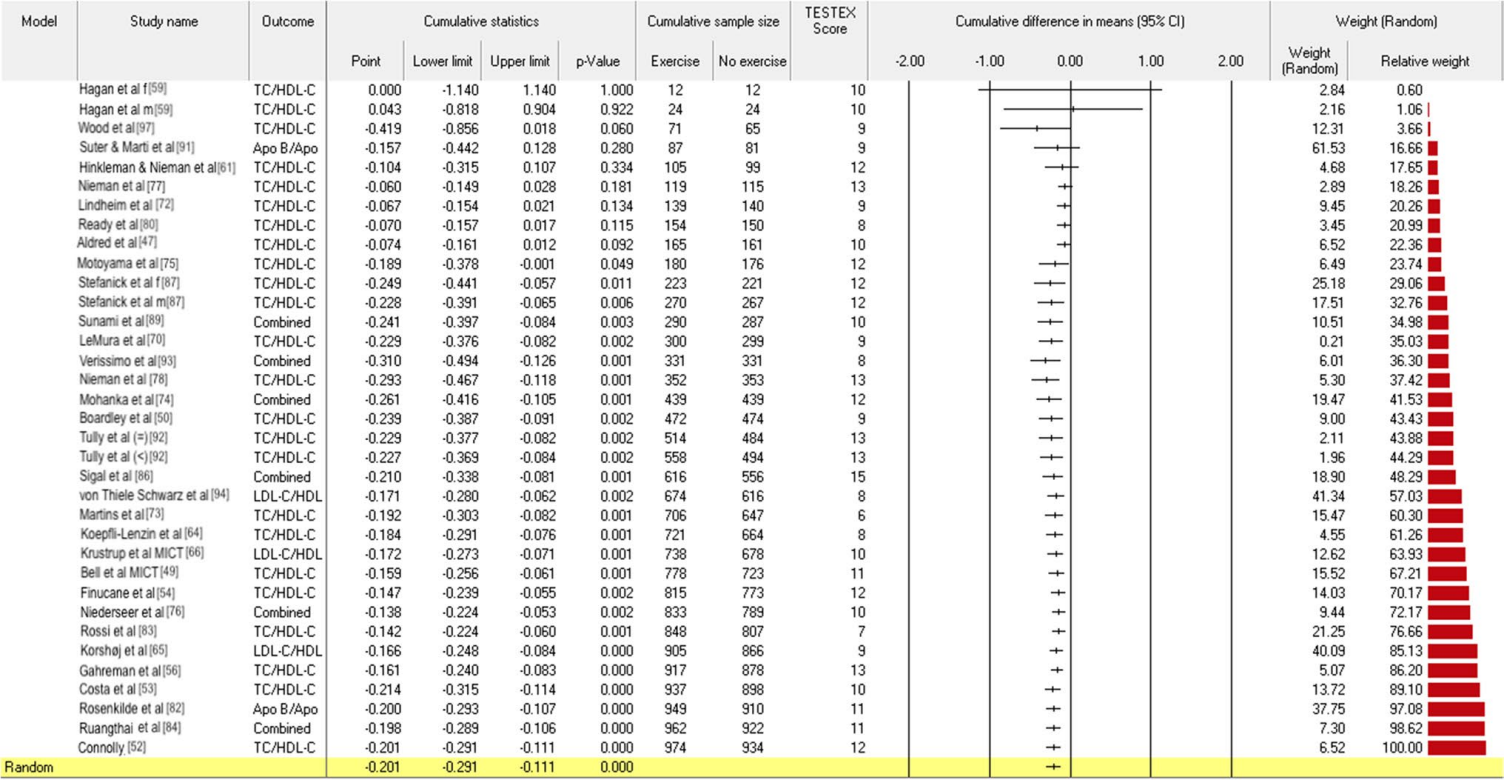
Joined TC/HDL-C, LDL-C/HDL-C ratio. *Apo* apolipoprotein, *CI* confidence interval, *Combined* joined outcomes, *f* females, *HDL-C* high-density lipoprotein cholesterol, *LDL-C* low-density lipoprotein cholesterol, *m* males, *MICT* moderate-intensite continuous training, *Random* random effects model, *TESTEX* study quality score (numeric), *TC* total cholesterol, (=) exercise protocol equivalent to recommended exercise levels, (<) exercise protocol less than recommended exercise levels

Antiatherogenic Apo A1 and A2, with or without the inclusion of HDL2 and HDL3, and independent of unit of measurement, were statistically significantly raised by AET, as shown in Fig. 2 (mmol/L MD 0.047 (95% CI 0.011, 0.082), *P* = 0.01) and Fig. 3 (mg/dL MD 2.297 (95% CI 0.441, 4.153), *P* < 0.02) below. The joined TC/HDL-C + LDL-C/HDL-C + Apo B100/Apo A1 ratio was reduced with AET by a statistically significant amount, as shown in Fig. 4 (MD − 0.201 (95% CI − 0.291, − 0.111), *P* < 0.001). Sub-analyses using *K* − 1 sensitivity analysis for statistically significant outcomes did not change the results; see Figs. S1–S3 (OSM).

### Study Quality and Reporting

**Fig. 5:**
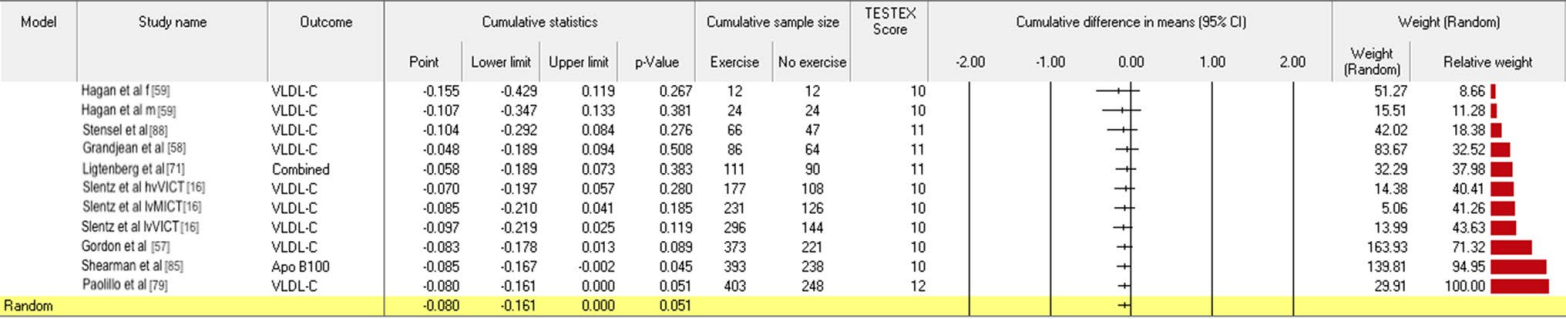
Atherogenic apolipoprotein and subfraction (joined B100 + VLDL), SQ ≥ 10. *Apo* apolipoprotein, *CI* confidence interval, *Combined* joined outcomes, *HDL-C* high-density lipoprotein cholesterol, *hvVICT* high-volume vigorous-intensity continuous training, *LDL-C* low-density lipoprotein cholesterol, *lvMICT* low-volume moderate-intensity continuous training, *lvVICT* low-volume vigorous-intensity continuous training, *Random* random effects model, *SQ* ≥10 study quality score greater than 10, *TESTEX* study quality score (numeric), *TC* total cholesterol, *VLDL-C* very-low-density lipoprotein cholesterol

The median study quality TESTEX score was 10 (from a maximum score of 15; range 6–15), see Table S5 (OSM). Within-study risk of bias was mainly low or medium, see Table S6 (OSM). No RCT attained a TESTEX score ≥ 10 with a high within-study risk of bias score. Sub-analyses using TESTEX scores ≥ 10 resulted in statistical significance for atherogenic Apo B100 combined with VLDL, as shown in Fig. 5 (mmol/L MD − 0.08 (95% CI − 0.161, 0.000), *P* = 0.05), and the TC/HDL-C + LDL-C/HDL-C + Apo B100/Apo A1 ratio remained statistically significant; see Table S7 (OSM). Better quality studies increased the effect size for Apo B100 reported in mg/dL but did not attain statistical significance (Table S7, OSM).

### Lipid Extraction Methodology

The included RCTs extracted blood from individuals in fasted states and in seated or supine positions and thus no RCT was excluded for phlebotomy reasons (data not shown). Lipoprotein and apolipoprotein analysis, although under-reported in detail, included density gradient ultracentrifugation; immunoturbidimetric methods (Roche) in serum; sodium dodecyl sulfate–polyacrylamide gel electrophoresis (SDS-PAGE), immunoprecipitation enhanced by polyethylene glycol, and nuclear magnet resonance spectroscopy; however, sensitivity analysis according to analytical method, when possible to perform, did not change the results (data not shown).

### Small Study Effects

Included studies exceeded the minimum number of effect sizes to be pooled [98]. There was minimal to no evidence of potential small study effects for each of the statistically significant outcomes after analysis with Classic fail-safe *N*, Orwin’s fail-safe *N*, Duval and Tweedie’s trim-and-fill, Egger’s regression test, and Begg and Mazumdar’s rank correlation test, nor following visual inspection of precision and standard error funnel plots. Given the minimal evidence, the impact of the potential small study effects is trivial, which suggests validation of the results of the corresponding multivariate meta-analyses; see Tables S8–S11, Figs. S4a–S7b (OSM).

### Heterogeneity

Neither the degree of absolute between-study heterogeneity (*τ*^2^) nor the relative heterogeneity (*I*^2^) for each analysed outcome indicated that trials should not be pooled, or that significance testing should not be undertaken; see Table S12 (OSM).

### Meta-Regression

Exploratory multivariate meta-regression modelling of significant results suggested an association between AET volume (intervention covariates: intensity, minutes per session, sessions per week, and intervention duration) and improvement in the joined TC/HDL-C + LDL-C/HDL-C + Apo B100/Apo A1 ratio (*τ*^2^ = 0.0023, *R*^2^ = 0.84); see Table S13a (OSM). The study covariate publication year was minimally associated with improvement in the joined TC/HDL-C + LDL-C/HDL-C + Apo B100/Apo A1 ratio (*τ*^2^ = 0.0134, *R*^2^ = 0.07); see Table S13b (OSM). Our maximum likehood modelling of intervention and study covariates, whether singly or combined, alluded to overfitting of the data for antiatherogenic outcome Apo A1 + Apo A2 + HDL2 + HDL3 mmol/L; see Tables S14a and b (OSM). Variations of the model (restricted maximum likelihood, method of moments) suggested no fit or overfitting, respectively (data not shown).

## Discussion

This systematic review and cumulative random multivariate meta-analysis, with meta-regression of 57 RCTs of 3194 participants, compared the effects of at least 12 weeks of AET performed at > 40% VO_2MAX_ against non-exercising control groups on lipoprotein sub-fractions, apolipoproteins, associated ratios and lipid ratios in sedentary adults free of chronic diseases other than the CVD risk factors comprising MetS. Despite the potential for calculating a smaller effect size and obtaining statistical insignificance by adopting a conservative multivariate meta-analytical approach, we have shown that AET at > 40% VO_2MAX_ for ≥ 12 weeks achieved better outcomes than no exercise for lipoprotein sub-fractions, lipid and Apo ratios, as well as Apo A1, A2 and B100, which appear to be superior predictors of CVD risk compared to the SLP [4, 5, 9–14]. These CVD risk biomarkers could potentially be prioritised for measurement over the SLP. Using the measurement of these CVD risk biomarkers, AET can be prescribed in sufficient quantities to reduce CVD risk. Such a shift reflects the increasing trend towards personalised medicine and availability of economical and advanced lipoprotein tests [99, 100].

Our work corroborates other investigations of intervention covariates that might explain favourable change in SLP biomarkers [19, 21, 45, 46]. We found that increasing AET volumes (intensity, frequency, duration and chronicity) are associated with a beneficial change in lipid ratios that are considered highly indicative of CVD risk. Our recent comparison of the effects of high-intensity versus moderate-intensity aerobic exercise on lipids suggested that antiatherogenic HDL-C is positively affected by high intensity more than moderate intensity exercise [101].

Our results suggest that the study covariate publication year is only minimally associated with improvement in lipid and apolipoprotein ratios. Advances in lipid and apolipoprotein extraction techniques and measurement, if represented by the study covariate publication year, may have a limited influence on the changes observed in these biomarkers as a result of AET. Extraction techniques and measurement would thus be unlikely to explain variation in results of individual RCTs investigating the effects of AET. Our sensitivity analysis using lipid extraction methodology and measurement did not change our results. None of our included RCTs reported aggregated baseline lipid values suggesting extreme dyslipidaemia, for which next-generation DNA-sequencing may be more diagnostically appropriate for lipid disorders [102], and no RCTs reported including this technique. Conversely, our study quality sensitivity analyses showed that atherogenic Apo B100 + VLDL mmol/L achieved a statistically significant effect size for RCTs with a study quality score ≥ 10. These results allude to improvements in study design over time, and/or the employment of more sensitive lipid assays, irrespective of study publication year.

### Clinical Significance and Future Research

Antiatherogenic apolipoproteins were statistically significantly increased with AET by 0.05 mmol/L or 2.30 mg/dL (grouped by unit of measurement). A one SD increment of Apo A1 is associated with a reduced hazard ratio of major cardiovascular events [103, 104]. While not statistically significant, for RCTs reporting Apo B100 with a study quality score ≥ 10, we showed that AET beneficially decreased Apo B100 by 2.073 mg/dL. Given that a 9% reduction in coronary heart disease occurs for every 10 mg/dL reduction in ApoB100 [105], we calculate that our estimated 2.073 mg/dL decrease in ApoB100 could anticipate a 4.34% decrease in coronary heart disease. For RCTs with a study quality score ≥ 10, AET reduced the combined biomarker Apo B100 + VLDL-C by the statistically significant amount of 0.08 mmol/L. Such a decrease in atherogenic lipoproteins and sub-fractions is also associated with a similar reduction in coronary heart disease [105–107]. Our results reinforce the prescription of AET as a central tool in lipid management.

Our exploratory meta-regression analysis indicated that AET intervention volume (intensity, session minutes, sessions per week, and intervention duration) contribute to positive change in these CVD risk biomarkers. Other studies have found that an AET protocol of at least 180 min per week at > 40% VO_2MAX_ o﻿r > 1200 kcal/week has a beneficial influence on lipids [45, 46, 108, 109]. A previous systematic review pooling data from AET interventions compared thresholds of intensity and volume, and determined that a minimum effective AET protocol required an AET volume of > 45 min per session for 3–4 sessions per week for a duration of > 26 weeks at > 65% VO_2MAX_ [19] to positively affect lipids. To obtain larger effects on both the lipid CVD risk biomarkers we measured and their associated reductions in CVD incidence, the volume and intensity of weekly AET may need to be increased above global guidelines of 150 min of moderate intensity AET or 75 min of vigorous intensity AET per week [110] to the thresholds described above. Clinicians profiling their patients with the lipid CVD risk biomarkers we measured can prescribe AET protocols that more closely align with the patient’s preference by varying the intervention covariates associated with change in these biomarkers. As a minimum, weekly AET volumes in excess of 180 min at upper moderate intensities (> 65% VO_2MAX_) should be targeted.

Given the paucity of apolipoprotein, sub-fraction and ratio data reported in exercise trials, we propose that future research should compare AET protocols of appropriate volume against non-exercising interventions and report apolipoproteins, lipoprotein sub-fractions and relevant ratios, including the atherogenic index of plasma (log ratio of TRG/HDL-C). Since TRG better predicts CVD risk in women [111], we recommend trials also record, and by sex, non-HDL-C, non-HDL-C/HDL-C and the log ratio of TRG/HDL-C, as these ratios were under-reported in our included RCTs. Additionally future trials could also explore the effect of acute and chronic AET protocols on these lipid biomarkers, with additional pharmacotherapeutic and dietary interventions for comparison, in sedentary adults achieving less than recommended exercise volumes, both with and free of chronic disease and/or MetS factors.

Our study quality TESTEX and within-study risk of bias analyses indicated that included RCTs failed to specify one or more of the following: method of randomisation and allocation concealment; medication use, drop-out reasons, or adverse events; monitoring of the non-exercising group or adherence to either the exercising or non-exercising protocol; a minimum compliance level; the use of objective measuring devices; and reporting of post-intervention exercise volume (total sessions attended, total minutes per session, achieved intensity). Phlebotomy specifics of post-intervention blood extraction methodology and analyses were not always recorded. Participant data for pre–post body weight, body fat or lean mass, waist circumference or BMI, systolic and diastolic blood pressure, and fasting blood glucose, were also missing. Researchers investigating the effects of AET can better report their findings by including quantitative data for these participant characteristics.

### Strengths and Limitations of this Systematic Review and Multivariate Meta-Analysis with Meta-Regression

Our work has a number of strengths. To the best of our knowledge, this quantitative review is the first to have compared the effects of a minimum prescribed AET dose against no exercise on lipid sub-fractions, ratios and apolipoproteins in a cohort characterised as free of chronic disease except for the possible presence of the CVD risk factors comprising MetS, using a multivariate meta-analytic approach.

Previous systematic reviews did not use TESTEX [39] to measure the quality of included studies. We followed a rigorous inclusion/exclusion protocol to ensure minimisation of confounding factors amongst the RCT populations [112].

A potential limitation of our work is the use of aggregated RCT data and not individual subject data [113, 114], with the exception of one study [86]. We searched using English language terms, potentially reducing the pool of available studies for selection and possibly introducing bias. We excluded studies with intervention and comparison group *N* < 10, and the number of RCTs included with longer durations were few, which may have reduced estimated effect sizes. We included AET protocols with an intensity (> 40% VO_2MAX_) at the lower end of the moderate-intensity range; this low-intensity may elicit very small changes in lipids [19], and the inclusion of these protocols may have resulted in understated effect sizes. Study reporting of protocol adherence and intensity varied. Some RCTs used objective measures such as electronic devices. Other studies used subjective measures, for example, the Borg scale, self-reported heart rate, log books, denoted by different indices of intensity (energy expenditure, VO_2MAX_, maximum heart rate, METs, Borg scale). Bias in the measurement of data reported in the included RCTs may have thus occurred. Some RCTs reported scant data on AET protocol or energy expenditure, and we therefore estimated intensity as a percent of VO_2MAX_. A very small number of RCTs noted that control groups increased physical activity levels during the duration of the study; this may have reduced our estimated effect size. Since our meta-regression covariates were not randomised at study level, our meta-regression findings are best viewed as exploratory. With respect to data pooling, where the standard deviation of the mean difference, or exact *P* values within groups, or 95% CIs were not available, statistical analyses depended on extrapolated data. Our imputation of these statistics was conservative and this approach may have weakened results.

## Conclusion

This multivariate meta-analysis with meta-regression of pooled data indicated that AET programs of moderate intensity with a minimum 12-week duration significantly reduced the joint TC/HDL-C, LDL-C/HDL-C and Apo B100/Apo A1 ratios, as well as Apo B100 and VLDL values, while significantly raising Apo A1 and A2 and the sub-fractions HDL2 and HDL3, in sedentary adults free of chronic disease other than possible MetS factors. Our results mimic the results of previous reviews examining standard lipid CVD risk biomarkers and our exploratory meta-regression suggested AET volume is associated with change in lipid ratios and antiatherogenic apolipoproteins and lipoprotein sub-fractions. Few studies reported the Apo B100/Apo A1 ratio, considered an equivalent if not more accurate lipid CVD risk biomarker in comparison to standard lipid CVD risk biomarkers. Aerobic exercise training has been shown to affect lipoprotein sub-fractions, ratios and apolipoproteins, and positive changes in these biomarkers are associated with reduction in CVD risk. Clinicians can confidently prescribe aerobic exercise training programs as part of overall CVD risk management and monitor the effect of these programs using these novel lipid biomarkers.

## Supplementary Information

Below is the link to the electronic supplementary material.

Supplementary file1 (DOCX 1514 KB)

## Declarations

### Code availability

The authors report that no code availability statement is applicable to this systematic review.

### Data availability

Data are available on request.
