# Supplementary material for: Estimating the Effect of Aerobic Exercise Training on Novel Lipid Biomarkers: A Systematic Review and Multivariate Meta-Analysis of Randomized Controlled Trials
Source: Sports Med. 2023 Mar 2;53(4):871–86. doi: 10.1007/s40279-023-01817-0 (PMC10036419; doi:10.1007/s40279-023-01817-0)
Supplement: Supplementary file 1 — Supplementary file1 (DOCX 1514 KB) [file 40279_2023_1817_MOESM1_ESM.docx]

**Supplementary Information**

**Measuring the effect of aerobic exercise training on novel lipid biomarkers: A systematic review and multivariate meta-analysis of randomised controlled trials.**

**Title:**

**Estimating the effect of aerobic exercise training on novel lipid biomarkers: a systematic review and multivariate meta-analysis of randomized controlled trials.**

**Running heading:**

**Aerobic exercise training and novel lipid biomarkers: A systematic review and multivariate meta-analysis.**

Gina Wood1 2*, Emily Taylor1, Vanessa Ng1, Anna Murrell3, Aditya Patil1, Tom van der Touw1, Mitch Wolden^4^, Nick Andronicos^1^, Neil A Smart1

* Corresponding author at:

1 School of Science and Technology in the Faculty of Science, Agriculture, Business and Law, University of New England, Armidale, NSW, Australia 2351.

Email: gnadinew@une.edu.au

ORCID: [0000-0002-5096-4989](http://orcid.org/0000-0002-5096-4989)

Emily Taylor ORCID: 0000-0001-8450-1125

Vanessa Ng ORCID: 0000-0001-7285-3916

Aditya Patil ORCID: 0000-0001-8215-5759

Tom van der Touw ORCID: 0000-0003-3507-1178

Nick Andronicos ORCID: 0000-0001-5881-2296

Neil A Smart ORCID: 0000-0002-8290-6409

^2^ School of Allied Health, Curtin University, Bentley, WA, Australia 6102

^3^ School of Rural Medicine in the Faculty of Medicine and Health, University of New England, Armidale, NSW, Australia 2351.

Anna Murrell ORCID: 0000-0002-3841-2305

^4^ Physical Therapy Program, University of Jamestown, Fargo, ND, USA 58104

Mitch Wolden ORCID: 0000-0002-9390-1590

.

**Table S1 Search Strategy example**

MeSH and free text terms such as aerobic exercise training, physical activity, endurance exercise, lipids, lipoproteins, apolipoproteins, triglycerides, and cholesterol were used. Searches excluded studies of pregnant or lactating females; elite athletes; juveniles; current or previous incidence of cardiovascular disease, stroke, cancer, non-alcoholic fatty liver disease populations; chronic disease; and dietary and pharmaceutical interventions.

| Web of Science example search | TOPIC:(random* control* trial*) *AND*  TOPIC:(*cholesterol*  OR *lipoprotein*  OR triglycer*  OR lipid*) *AND*  TOPIC:(exercise  OR physical activity  OR aerobic training  OR moderate intensity  OR high intensity  OR HIIT  OR MICT OR endurance)  *NOT* TOPIC: (heart failure  OR belief*  OR *statin*  OR diet*  OR HIV  OR cardiac rehabilitation  OR NAFLD  OR *Alzheimer*  OR *stroke  OR cancer  OR athlete  OR child*  OR pregnan* or lactat* or adolescent  OR juvenile OR bariatric OR renal failure OR polycystic OR depression)  *NOT* TOPIC:(systematic review*  OR meta-analys*)  Timespan: All years.  Databases:  WOS, CABI, CCC, KJD, MEDLINE, RSCI, SCIELO.  Search language=Auto |
| --- | --- |

**Table S2 Intervention inclusion and exclusion criteria**

| **Exclusion criteria** | Studies using either an isometric, unconventional, resistance- or combined-training intervention, without separate AET interventions as comparators against a non-exercising control group, were excluded.  Studies using lifestyle, dietary or pharmaceutical interventions, without separate AET interventions as comparators against a non-exercising control group, were excluded.  Studies comparing multiple AET protocols without a non-exercising control group as comparator were excluded.  Studies which did not provide details of the AET protocol, such as session duration, intensity, number of sessions in the intervention, or other details which allowed estimation of volume of exercise if not reported, were excluded. |
| --- | --- |

AET: aerobic exercise training

**Table S3 TESTEX Assessment of Study Quality**

One point is awarded per criterion, for a total maximum of 15 points.

| Author Year | Eligibility criteria specified | Random-isation specified | Allocation concealment | Groups similar at baseline | Blinding of assessor | Outcomes measures assessed in 85% patients | Adverse events reported | Exercise adherence reported | Intention-to-treat analysis | Between-group statistical comparisons reported for primary outcome | Between-group statistical comparisons reported for secondary outcome | Point measures and measures of variability for all outcome measures | Activity monitoring in control groups | Relative exercise intensity remained constant | Exercise volume and energy expenditure given | Overall TESTEX  (/15) |
| --- | --- | --- | --- | --- | --- | --- | --- | --- | --- | --- | --- | --- | --- | --- | --- | --- |
| Author Name, Year | 0 | 1 |  |  |  |  |  |  |  |  |  |  |  |  |  | **=** |

**Table S4 Assessed Within-Study Risk of Bias Factors**

| Author Year | Study non-randomised or randomised | Minimum compliance level set | Habitual medication use reported | Dropout reason reported | Baseline fitness and effort determined | > 50% sessions supervised | Effort monitoring and measurement device | Risk of bias assesment low, medium, or high |
| --- | --- | --- | --- | --- | --- | --- | --- | --- |
| Author, Year |  |  |  |  |  |  |  |  |

Either of low or high awarded as follows:

1. Study non-randomised or randomised – low if randomised, high if non-randomised;^1^
2. For intervention groups, a minimum level of compliance to be counted as having participated in the intervention group or control group – low if a minimum level of compliance was set or reported, high if there was no minimum compliance level;
3. Habitual medication use reported – low if reported, high if not reported;
4. Drop-out reasons given – low if reported, high if not reported;
5. Baseline fitness and effort determined – low if baseline fitness and effort was measured, high if not determined;
6. > 50% of sessions supervised – low if > 50% of sessions were supervised, high if not; and
7. Effort monitoring and measurement devices – low if digital recording devices were used, high if analog or no device.

Studies were scored overall low, medium, or high risk of bias according to the number of times either “low” or “high” was accorded. A low risk of bias was awarded for 0-2 instances of “high”, a medium risk of bias was awarded for 3-4 instances of “high”, and a high risk of bias was awarded for 5-7 instances of “high”. All factors were equally weighted.

^1^ All studies were randomised

**Sensitivity Analyses (K-1 sub-analysis)**

**Fig. S1 Random multivariate meta-analysis of joined outcomes Apo A1 + Apo A2 + HDL2 + HDL3 mmol/L with one study removed per line**

**
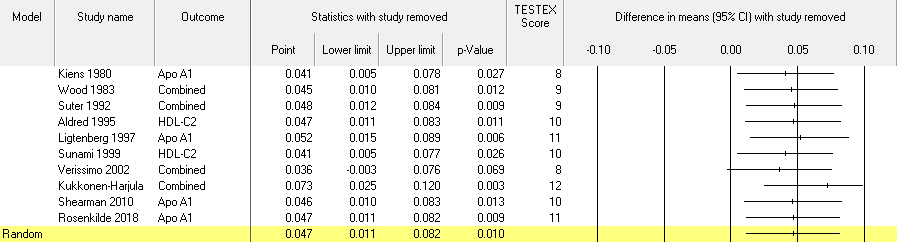
**

Random: random effects model; TESTEX: study quality score (numeric); CI: confidence interval; Apo: apolipoprotein; HDL-C: high-density lipoprotein cholesterol; Combined: joined outcomes.

**Fig. S2 Random multivariate meta-analysis of joined outcomes Apo A1 + Apo A2 mg/dL with one study removed per line**

**
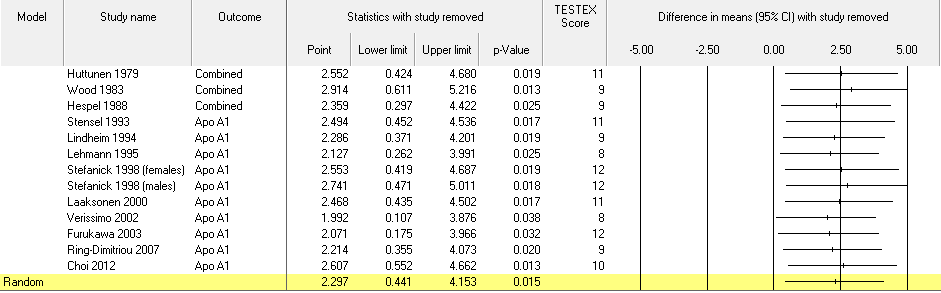
**

Random: random effects; TESTEX: study quality score (numeric); CI: confidence interval; Apo: apolipoprotein; Combined: joined outcomes.

**Fig. S3 Random multivariate meta-analysis of joined outcomes TC/HDL-C + LDL-C/HDL-C + Apo B100/Apo A1 with one study removed per line**

**
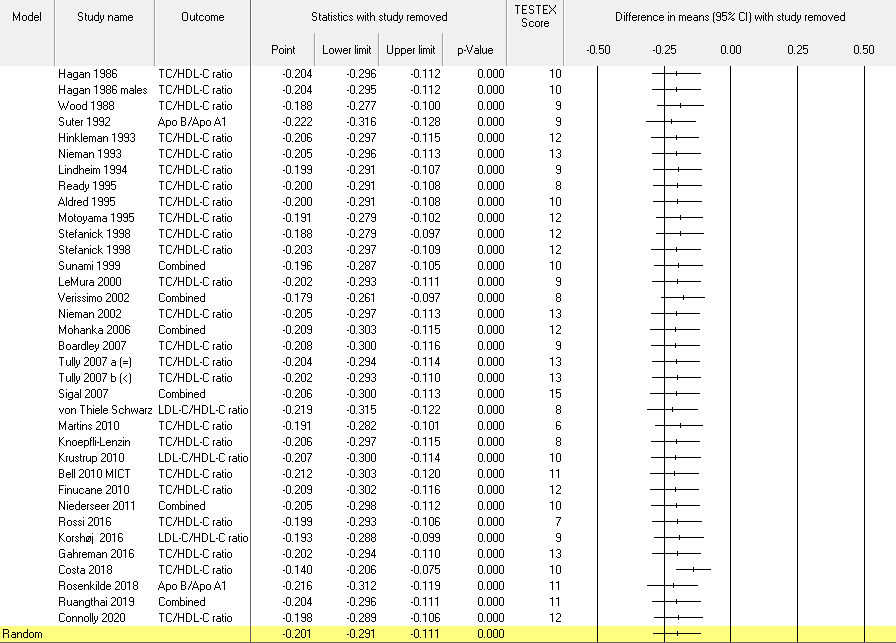
**

Random: random effects; TESTEX: study quality score (numeric); CI: confidence interval; Apo: apolipoprotein; TC: total cholesterol; HDL-C: high-density lipoprotein cholesterol; LDL-C: low-density lipoprotein cholesterol; Combined: joined outcomes; (=): exercise protocol equivalent to recommended exercise levels; (<): exercise protocol less than recommended exercise levels

**Table S5 TESTEX Assessment of Study Quality**

| Author Year | Eligibility criteria specified | Random-isation specified | Allocation conceal-ment | Groups similar at baseline | Blinding of assessor | Outcomes measures assessed in 85% patients | Adverse events reported | Exercise adherence reported | Intention-to-treat analysis | Between-group statistical comparisons reported for primary outcome | Between-group statistical comparisons reported for secondary outcome | Point measures and measures of variability for all outcome measures | Activity monitoring in control groups | Relative exercise intensity remained constant | Exercise volume and energy expenditure given | Overall TESTEX  (/15) |
| --- | --- | --- | --- | --- | --- | --- | --- | --- | --- | --- | --- | --- | --- | --- | --- | --- |
| Aldred 1995 | 1 | 0 | 0 | 1 | 1 | 1 | 0 | 1 | 0 | 1 | 1 | 1 | 0 | 1 | 1 | **10** |
| Baker 1986 | 1 | 0 | 0 | 1 | 1 | 1 | 0 | 1 | 0 | 1 | 1 | 1 | 0 | 1 | 0 | **9** |
| Bell 2010 | 1 | 0 | 1 | 1 | 1 | 0 | 0 | 1 | 0 | 1 | 1 | 1 | 1 | 1 | 1 | **11** |
| Boardley 2007 | 1 | 0 | 0 | 1 | 1 | 1 | 0 | 1 | 0 | 1 | 1 | 1 | 0 | 1 | 0 | **9** |
| Choi 2012 | 1 | 1 | 0 | 1 | 1 | 1 | 0 | 0 | 0 | 1 | 0 | 1 | 1 | 1 | 1 | **10** |
| Connolly 2020 | 1 | 1 | 1 | 1 | 1 | 0 | 1 | 1 | 0 | 1 | 1 | 1 | 0 | 1 | 1 | **12** |
| Costa 2018 | 1 | 1 | 1 | 1 | 1 | 0 | 0 | 1 | 1 | 1 | 1 | 1 | 0 | 0 | 0 | 10 |
| Finucane 2010 | 1 | 1 | 0 | 1 | 1 | 1 | 1 | 1 | 0 | 1 | 1 | 1 | 0 | 1 | 1 | **12** |
| Furukawa 2010 | 1 | 1 | 1 | 0 | 1 | 1 | 0 | 1 | 1 | 1 | 1 | 1 | 1 | 0 | 1 | **12** |
| Gahreman 2016 | 1 | 1 | 1 | 1 | 1 | 1 | 1 | 1 | 0 | 1 | 1 | 1 | 0 | 1 | 1 | **13** |
| Gordon 2008 | 1 | 0 | 0 | 1 | 1 | 1 | 0 | 1 | 0 | 1 | 1 | 1 | 0 | 1 | 1 | **10** |
| Grandjean 1996 | 1 | 0 | 1 | 1 | 1 | 1 | 0 | 0 | 1 | 1 | 1 | 1 | 0 | 1 | 1 | **11** |
| Hagan 1986 | 1 | 0 | 0 | 1 | 1 | 1 | 0 | 1 | 0 | 1 | 1 | 1 | 0 | 1 | 1 | **10** |
| Hespel 1988 | 1 | 0 | 0 | 1 | 1 | 1 | 0 | 1 | 1 | 1 | 1 | 1 | 0 | 0 | 0 | **9** |
| Hinkleman 1993 | 1 | 1 | 1 | 0 | 1 | 1 | 1 | 0 | 0 | 1 | 1 | 1 | 1 | 1 | 1 | **12** |
| Huttunen 1979 | 1 | 0 | 1 | 1 | 1 | 1 | 0 | 1 | 0 | 1 | 1 | 1 | 0 | 1 | 1 | **11** |
| Kiens 1980 | 1 | 1 | 0 | 0 | 1 | 0 | 0 | 1 | 0 | 1 | 1 | 1 | 0 | 0 | 1 | **8** |
| Knoepfli-Lenzin 2010 | 1 | 0 | 0 | 0 | 1 | 0 | 1 | 1 | 0 | 1 | 1 | 1 | 0 | 0 | 1 | **8** |
| Korshøj  2016 | 1 | 1 | 0 | 1 | 1 | 0 | 0 | 0 | 1 | 1 | 1 | 1 | 0 | 0 | 1 | **9** |
| Krustrup 2010 | 1 | 0 | 0 | 1 | 1 | 0 | 1 | 1 | 0 | 1 | 1 | 1 | 0 | 1 | 1 | **10** |
| Kukkonen-Harjula 1998 | 1 | 1 | 0 | 1 | 1 | 1 | 1 | 1 | 1 | 1 | 1 | 0 | 0 | 1 | 1 | **12** |
| Laaksonen 2000 | 1 | 1 | 1 | 1 | 1 | 0 | 0 | 1 | 0 | 1 | 1 | 1 | 0 | 1 | 1 | **11** |
| Lehmann 1995 | 1 | 0 | 0 | 1 | 1 | 1 | 0 | 0 | 0 | 0 | 1 | 1 | 0 | 1 | 1 | **8** |
| LeMura 2000 | 0 | 1 | 0 | 0 | 1 | 1 | 0 | 0 | 0 | 1 | 1 | 1 | 1 | 1 | 1 | **9** |
| Ligtenberg 1997 | 1 | 0 | 0 | 1 | 1 | 1 | 1 | 1 | 0 | 1 | 1 | 1 | 0 | 1 | 1 | **11** |
| Lindheim 1994 | 1 | 0 | 0 | 0 | 1 | 1 | 0 | 0 | 1 | 0 | 1 | 1 | 1 | 1 | 1 | **9** |
| Martins 2010 | 1 | 0 | 0 | 0 | 1 | 0 | 0 | 0 | 0 | 1 | 1 | 1 | 0 | 0 | 1 | **6** |
| Mohanka 2006 | 1 | 1 | 0 | 1 | 1 | 1 | 0 | 1 | 1 | 1 | 1 | 1 | 1 | 0 | 1 | **12** |
| Motoyama 1995 | 1 | 1 | 0 | 1 | 1 | 1 | 0 | 1 | 1 | 1 | 1 | 1 | 0 | 1 | 1 | **12** |
| Niederseer 2011 | 1 | 0 | 0 | 1 | 1 | 0 | 1 | 1 | 0 | 1 | 1 | 1 | 0 | 1 | 1 | **10** |
| Nieman 1993 | 1 | 0 | 0 | 1 | 1 | 1 | 1 | 1 | 1 | 1 | 1 | 1 | 1 | 1 | 1 | **13** |
| Nieman 2002 | 1 | 0 | 1 | 1 | 1 | 1 | 0 | 1 | 1 | 1 | 1 | 1 | 1 | 1 | 1 | **13** |
| Paolillo 2017 | 1 | 1 | 1 | 1 | 1 | 0 | 1 | 0 | 0 | 1 | 1 | 1 | 1 | 1 | 1 | **12** |
| Ready 1995 | 1 | 0 | 0 | 0 | 1 | 0 | 0 | 1 | 0 | 1 | 1 | 1 | 0 | 1 | 1 | **8** |
| Ring-Dimitriou 2007 | 1 | 0 | 0 | 1 | 1 | 0 | 1 | 1 | 0 | 1 | 1 | 1 | 0 | 1 | 0 | **9** |
| Rosenkilde 2018 | 1 | 1 | 0 | 1 | 1 | 0 | 1 | 1 | 0 | 1 | 1 | 1 | 0 | 1 | 1 | **11** |
| Rossi 2016 | 1 | 0 | 0 | 0 | 1 | 0 | 0 | 0 | 0 | 1 | 1 | 1 | 0 | 1 | 1 | **7** |
| Ruangthai 2019 | 1 | 0 | 1 | 1 | 1 | 0 | 1 | 1 | 0 | 1 | 1 | 1 | 0 | 1 | 1 | **11** |
| Shearman 2010 | 1 | 0 | 0 | 1 | 1 | 1 | 0 | 0 | 0 | 1 | 1 | 1 | 1 | 1 | 1 | **10** |
| Sigal 2007 | 1 | 1 | 1 | 1 | 1 | 1 | 1 | 1 | 1 | 1 | 1 | 1 | 1 | 1 | 1 | **15** |
| Slentz 2007 | 1 | 0 | 1 | 1 | 1 | 1 | 0 | 1 | 0 | 1 | 0 | 1 | 0 | 1 | 1 | **10** |
| Stefanick 1998 | 1 | 1 | 1 | 1 | 1 | 1 | 0 | 1 | 0 | 1 | 1 | 1 | 0 | 1 | 1 | **12** |
| Stensel 1993 | 1 | 0 | 1 | 0 | 1 | 1 | 1 | 1 | 0 | 1 | 1 | 1 | 0 | 1 | 1 | **11** |
| Sunami 1999 | 1 | 0 | 0 | 1 | 1 | 1 | 0 | 1 | 0 | 1 | 1 | 1 | 0 | 1 | 1 | **10** |
| Suter 1990 | 1 | 0 | 0 | 1 | 1 | 1 | 0 | 1 | 0 | 1 | 1 | 0 | 0 | 1 | 1 | **9** |
| Suter 1992 | 1 | 0 | 0 | 1 | 1 | 1 | 0 | 1 | 0 | 1 | 1 | 0 | 0 | 1 | 1 | **9** |
| Tully 2007 | 1 | 1 | 0 | 1 | 1 | 1 | 1 | 1 | 1 | 1 | 1 | 1 | 1 | 0 | 1 | **13** |
| Verissimo 2002 | 1 | 0 | 0 | 1 | 1 | 1 | 1 | 0 | 0 | 1 | 0 | 0 | 0 | 1 | 1 | **8** |
| Von Thiele Schwarz 2008 | 1 | 0 | 0 | 1 | 1 | 1 | 0 | 0 | 0 | 1 | 1 | 0 | 0 | 1 | 1 | **8** |
| Wirth 1985 | 1 | 0 | 0 | 1 | 1 | 1 | 1 | 0 | 0 | 1 | 1 | 1 | 0 | 0 | 0 | **8** |
| Wood 1983 | 1 | 1 | 0 | 1 | 1 | 1 | 0 | 0 | 0 | 1 | 1 | 1 | 0 | 0 | 1 | **9** |
| Wood 1988 | 1 | 0 | 0 | 1 | 1 | 0 | 0 | 0 | 0 | 1 | 1 | 1 | 1 | 1 | 1 | **9** |

**Table S6 Assessed Within-Study Risk of Bias Factors**

| Author Year | Study non-randomised or randomised | Minimum compliance level set | Habitual medication use reported | Dropout reason reported | Baseline fitness and effort determined | > 50% sessions supervised | Effort monitoring and measurement device | Risk of bias assesment low, medium, or high |
| --- | --- | --- | --- | --- | --- | --- | --- | --- |
| Aldred 1995 | low | low | low | low | low | high | high | **low** |
| Baker 1986 | low | low | low | low | low | low | high | **low** |
| Bell 2010 | low | low | low | low | low | low | low | **low** |
| Boardley 2007 | low | low | low | high | high | low | high | **medium** |
| Choi 2012 | low | high | low | high | low | high | low | **medium** |
| Connolly 2020 | low | low | low | low | low | high | low | **low** |
| Costa 2018 | low | high | low | low | low | high | high | **medium** |
| Finucane 2010 | low | low | low | low | low | low | low | **low** |
| Furukawa 2003 | low | high | high | low | low | high | low | **medium** |
| Gahreman 2016 | low | high | low | low | low | low | **low** | low |
| Gordon 2008 | low | low | high | high | low | high | high | **medium** |
| Grandjean 1996 | low | low | high | high | low | high | high | **medium** |
| Hagan 1986 | low | low | high | high | low | low | high | **medium** |
| Hespel 1988 | low | low | low | low | low | low | high | **low** |
| Hinkleman 1993 | low | high | low | low | low | low | low | **low** |
| Huttunen 1979 | low | high | low | low | low | high | high | **medium** |
| Kiens 1980 | low | high | high | high | high | high | low | **high** |
| Knoepfli-Lenzin 2010 | low | low | high | low | low | low | low | **low** |
| Korshøj  2016 | low | high | high | high | low | low | low | **medium** |
| Krustrup 2010 | low | low | low | low | low | low | low | **low** |
| Kukkonen-Harjula 1998 | low | low | low | low | low | low | low | **low** |
| Laaksonen 2000 | low | high | low | low | low | low | high | **low** |
| Lehmann 1995 | low | low | low | low | low | high | high | **low** |
| LeMura 2000 | low | low | high | high | low | high | low | **medium** |
| Ligtenberg 1997 | low | low | low | low | low | high | high | **low** |
| Mohanka 2006 | low | low | low | high | low | high | low | **low** |
| Motoyama 1995 | low | high | low | low | low | low | high | **low** |
| Niederseer 2011 | low | high | low | high | low | low | low | **low** |
| Nieman 1993 | low | low | low | low | low | low | low | **low** |
| Nieman 2002 | low | low | high | low | low | low | low | **low** |
| Paolillo 2017 | low | high | high | low | low | low | low | **low** |
| Ready 1995 | low | low | high | low | low | high | high | **medium** |
| Ring-Dimitriou 2007 | low | high | high | low | low | low | high | **medium** |
| Rosenkilde 2018 | low | low | high | low | low | low | low | **low** |
| Rossi 2016 | low | low | high | low | high | high | high | **medium** |
| Ruangthai 2019 | low | low | low | low | low | low | low | **low** |
| Shearman 2010 | low | high | low | low | low | high | high | **medium** |
| Sigal 2007 | low | low | low | low | low | low | low | **low** |
| Slentz 2007 | low | low | high | high | low | low | low | **low** |
| Stefanick 1998 | low | high | high | high | low | low | high | **medium** |
| Stensel 1995 | low | high | low | low | low | high | low | **low** |
| Sunami 1999 | low | low | high | high | low | low | high | **medium** |
| Suter 1990 | low | low | high | high | low | high | low | **medium** |
| Suter 1992 | low | low | high | high | low | high | low | **medium** |
| Tully 2007 | low | high | high | low | low | high | high | **medium** |
| Verissimo 2002 | low | high | high | low | low | low | high | **medium** |
| von Thiele Schwarz 2008 | low | low | high | low | low | low | high | **low** |
| Wirth 1985 | low | high | high | low | low | low | high | **medium** |
| Wood 1983 | low | low | high | low | low | low | high | **low** |
| Wood 1988 | low | low | high | low | low | high | high | **medium** |

We awarded either of low or high for the following factors:

1. Study non-randomised or randomised – low if randomised, high if non-randomised;^1^
2. For intervention groups, a minimum level of compliance to be counted as having participated in the intervention group or control group – low if a minimum level of compliance was set or reported, high if there was no minimum compliance level;
3. Habitual medication use reported – low if reported, high if not reported;
4. Drop-out reasons given – low if reported, high if not reported;
5. Baseline fitness and effort determined – low if baseline fitness and effort was measured, high if not determined;
6. > 50% of sessions supervised – low if > 50% of sessions were supervised, high if not; and
7. Effort monitoring and measurement devices – low if digital recording devices were used, high if analog or no device.

Studies were scored overall low, medium, or high risk of bias according to the number of times either “low” or “high” was accorded. A low risk of bias was awarded for 0-2 instances of “high”, a medium risk of bias was awarded for 3-4 instances of “high”, and a high risk of bias was awarded for 5-7 instances of “high”. All factors were equally weighted.

^1^ All studies were randomised

**Table S7 Multivariate and Univariate Random Effects Meta-analysis Summary Statistics per Lipid Outcome, Study Quality ≥10**

| **Multivariate Analysis Model** | **Random, 95% CI, Maximum Likelihood, Knapp-Hartung, Mean Difference** | | | | | **Population N** | | |
| --- | --- | --- | --- | --- | --- | --- | --- | --- |
| **Apolipoprotein, sub-fraction, ratio**  **Mean of combined outcomes** | **Point Estimate** | | **Lower Limit** | **Upper Limit** | **p-value** | **Exercise** | **No Exercise** | **Total** |
| Apo A1 + Apo A2 + HDL2 + HDL3 mmol/L SQ | 0.027 | | -0.015 | 0.070 | 0.208 | 141 | 140 | 281 |
| Apo A1 + Apo A2 mg/dL SQ | 1.775 | | -0.725 | 4.275 | 0.164 | 255 | 243 | 498 |
| Apo B100 + VLDL mmol/L SQ | -0.080 | | -0.161 | 0.000 | **0.051** | 403 | 248 | 651 |
| TC/HDL-C + LDL-C/HDL-C + Apo B100/Apo A1 SQ | -0.192 | | -0.310 | -0.075 | **0.001** | 625 | 578 | 1203 |
| HDL-C/TC + HDL-C/LDL-C + Apo A1/Apo B100 SQ (Shearman 2010)) | | 0.100 | -0.145 | 0.345 | 0.423 | 20 | 17 | 37 |
|  |  | |  |  |  |  |  |  |
| **Univariate Analysis Model** | **Random, 95% CI, Maximum Likelihood, Knapp-Hartung, Mean Difference** | | | | | **Population N** | | |
| **Apolipoprotein, sub-fraction, ratio** | **Point Estimate** | | **Lower Limit** | **Upper Limit** | **p-value** | **Exercise** | **No Exercise** | **Total** |
| Apo B100 mg/dL SQ | -2.073 | | -4.896 | 0.750 | 0.150 | 211 | 197 | 408 |

Apo: apolipoprotein; CI: confidence interval, HDL/HDL-C: high-density lipoprotein(-cholesterol); N: sample size per group and total; Random: random effects model; SQ: study quality; TC: total cholesterol: VLDL: very low-density lipoprotein cholesterol;

**Small Study Effects**

1. **Apo A1 + Apo A2 + HDL2 + HDL3 mmol/L**
   1. **S8a. Classic fail-safe N and Orwin’s fail-safe N**
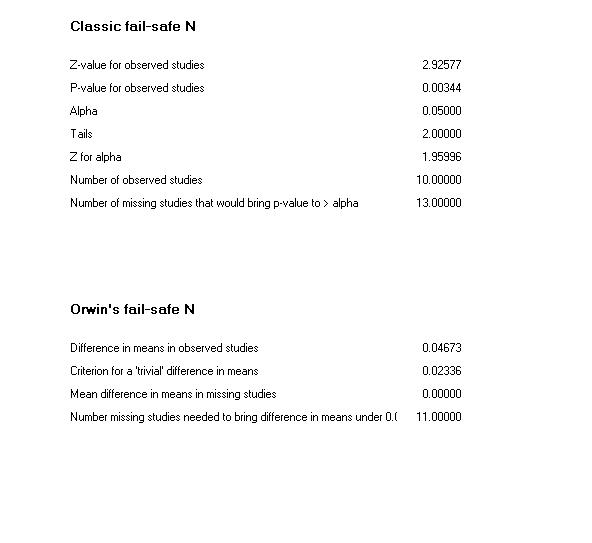

   2. **S8b. Begg and Mazumdar rank correlation**
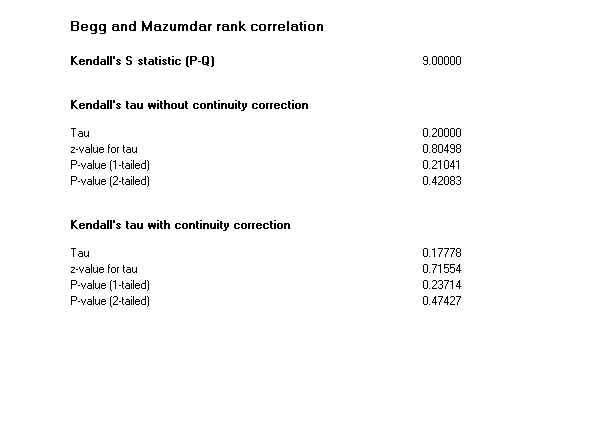

   3. **S8c. Egger’s regression intercept**
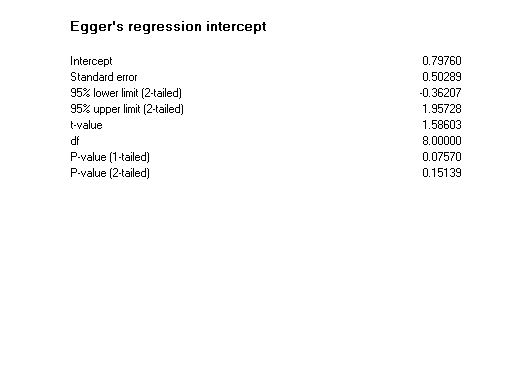

   4. **S8d. Duval and Tweedie’s trim and fill**
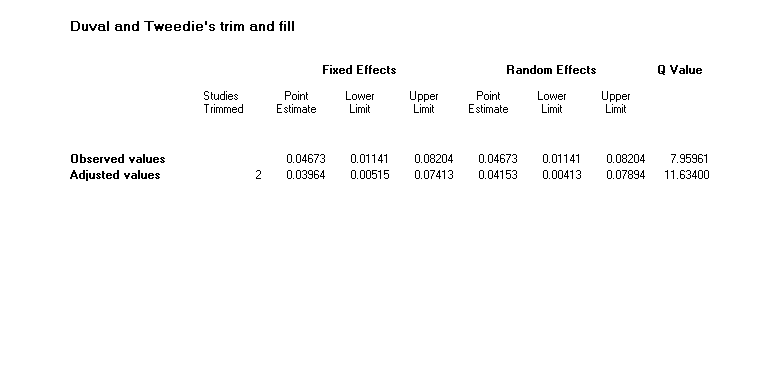


**Fig. S4a. Funnel Plot of Precision by Difference in Means (random effects)**

**Fig. S4b. Funnel Plot of Standard Error by Difference in Means (random effects)**

1. **Apo A1 + Apo A2 mg/dL**
   1. **S9a. Classic fail-safe N and Orwin’s fail-safe N
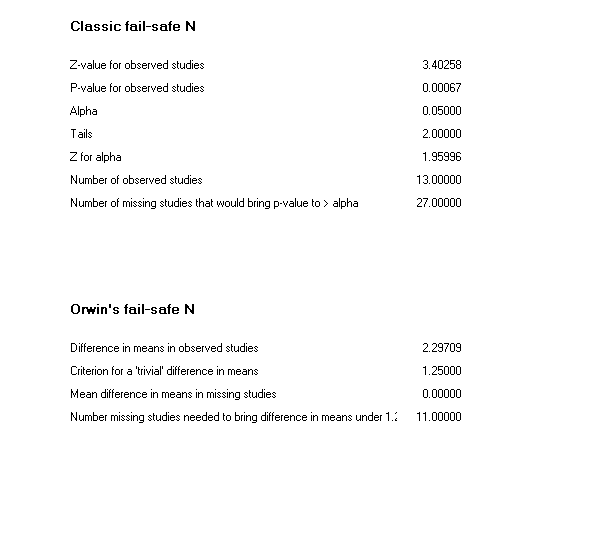
**
   2. **S9b. Begg and Mazumdar rank correlation
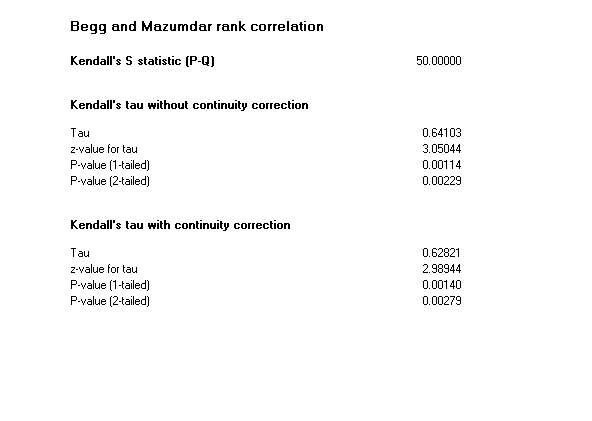
**
   3. **S9c. Egger’s regression intercept**


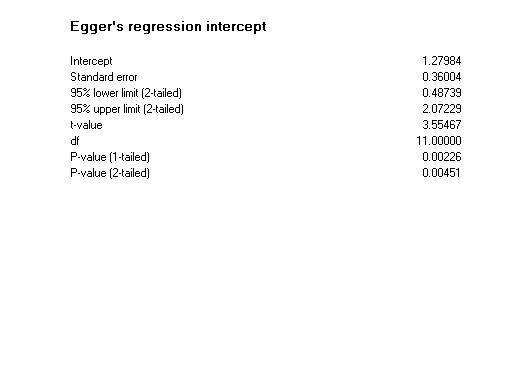


- 1. **S9d. Duval and Tweedie’s trim and fill
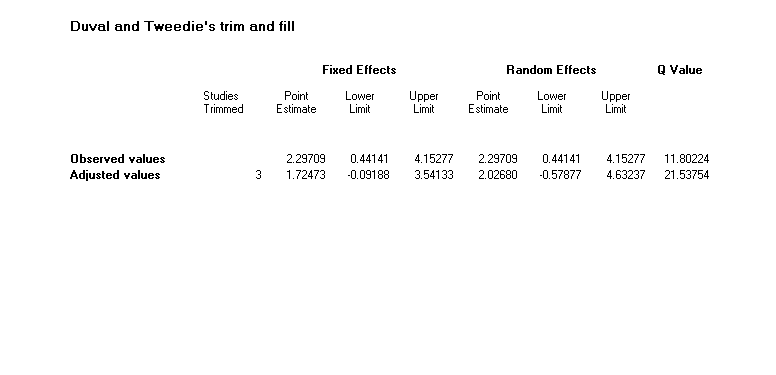
**

**Fig. S5a. Funnel Plot of Precision by Difference in Means (random effects)**

**Fig. S5b. Funnel Plot of Standard Error by Difference in Means (random effects)**

1. **Apo B100 + VLDL-C mmol/L SQ**
   1. **S10a. Classic fail-safe N and Orwin’s fail-safe N** **
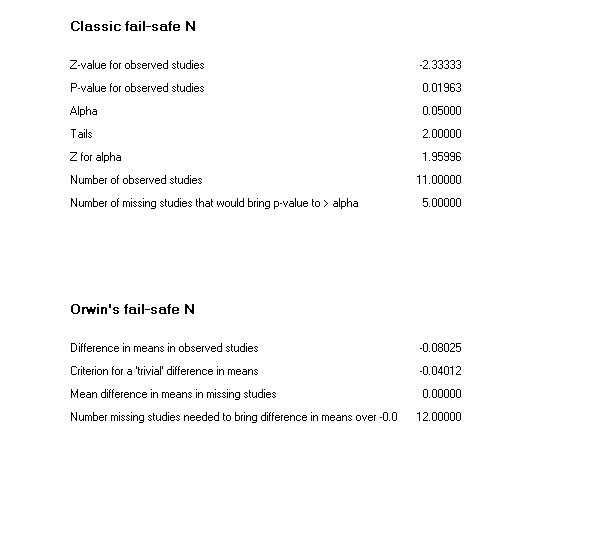
**
   2. **S10b.Begg and Mazumdar rank correlation** **
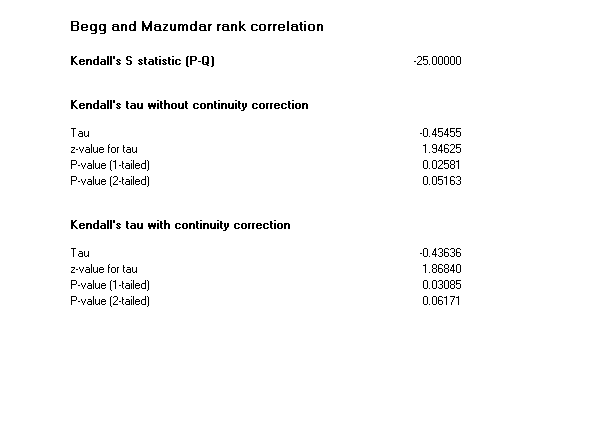
**
   3. **S10c. Egger’s regression intercept** **
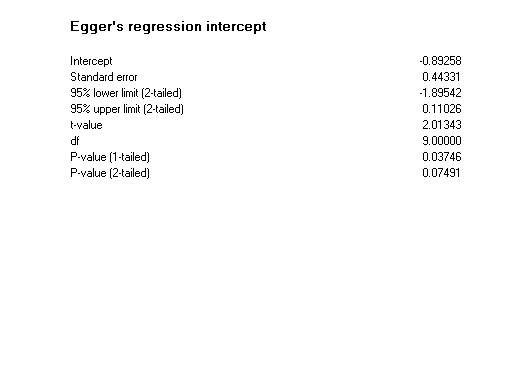
**
   4. **S10d. Duval and Tweedie’s trim and fill** **
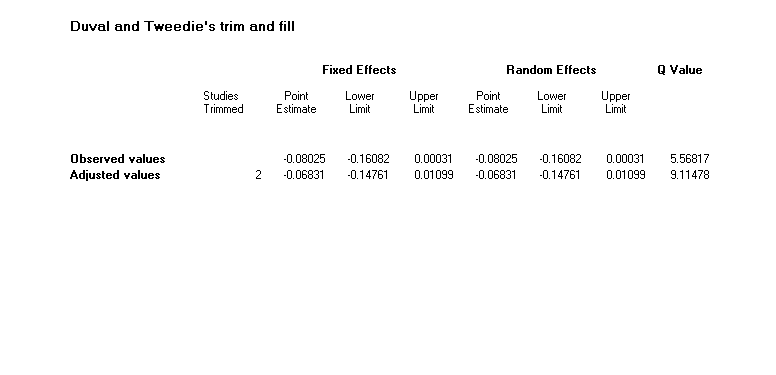
**

**Fig. S6a. Funnel Plot of Precision by Difference in Means (random effects)**

**Figure S6b. Funnel Plot of Standard Error by Difference in Means (random effects)**

1. **TC/HDL-C + LDL-C/HDL-C + Apo B100/Apo A1**
   1. **S11a. Classic fail-safe N and Orwin’s fail-safe N
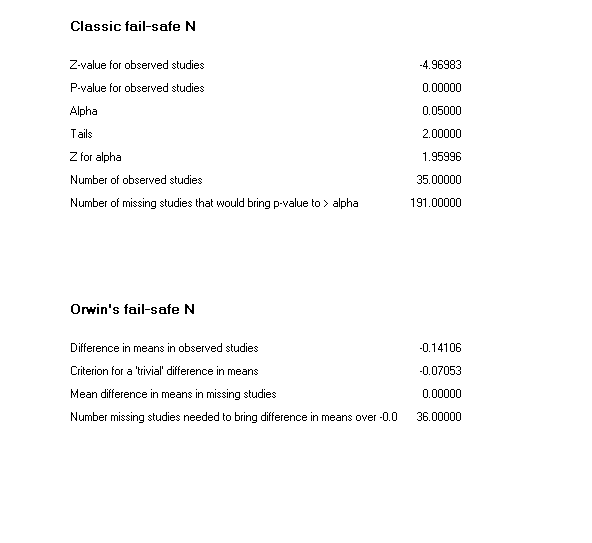
**
   2. **S11b. Begg and Mazumdar rank correlation
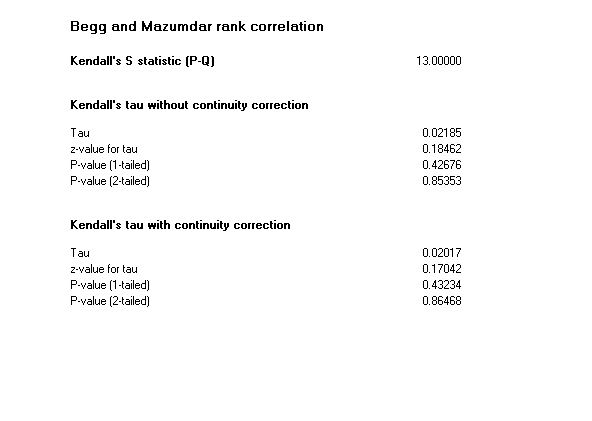
**
   3. **S11c. Egger’s regression intercept
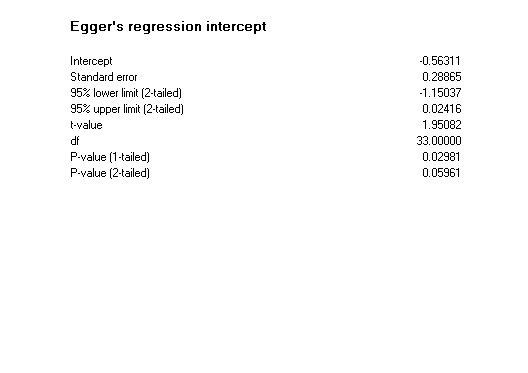
**
   4. **S11d. Duval and Tweedie’s trim and fill
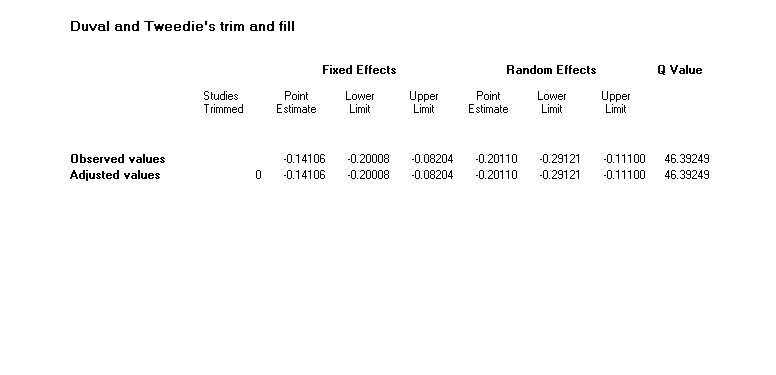
**

**Fig. S7a. Funnel Plot of Precision by Difference in Means (random effects)**

**Fig. S7b. Funnel Plot of Standard Error by Difference in Means (random effects)**

**Table S12 Heterogeneity reporting for I^2^ and τ^2^**

| **Outcome** | **Hetereogeneity** | | | | **τ^2^** | | | |
| --- | --- | --- | --- | --- | --- | --- | --- | --- |
|  | **Q-value** | **Df [Q]** | ***P***  **value** | **I^2^ %** | **τ^2^** | **Standard Error** | **Variance** | **τ** |
| Apo A1 + Apo A2 + HDL2 + HDL3 mmol/L | 7.96 | 9 | 0.54 | 0.00 | 0.00 | 0.00 | 0.00 | 0.01 |
| Apo A1 + Apo A2 mg/dL | 11.82 | 12 | 0.46 | 0.00 | 0.00 | 5.48 | 30.01 | 0.00 |
| TC/HDLC + LDL-C/HDL-C + Apo B100/ Apo A1 | 46.39 | 34 | 0.08 | 26.71 | 0.01 | 0.01 | 0.00 | 0.12 |
| HDL-C/TC + HDL-C/LDL-C + Apo A1/Apo B100 | 1.6 | 4 | 0.81 | 0.00 | 0.00 | 0.00 | 0.00 | 0.00 |
| Apo B100 + VLDL mmol/L | 7.42 | 16 | 0.96 | 0.00 | 0.00 | 0.01 | 0.00 | 0.00 |
| Apo B100 mg/dL | 9.92 | 12 | 0.62 | 0.00 | 0.00 | 4.99 | 24.89 | 0.00 |

Apo: apolipoprotein; HDL-C: high-density lipoprotein cholesterol; mmol/L: millimoles per litre; mg/dL: milligram per decilitre; VLDL: very low-density lipoprotein; TC: total cholesterol.

**Meta-regression Analyses**

**Table S13a TC/HDL-C + LDL-C/HDL-C + Apo B100/Apo A1 (intervention variables)**

| **Increments for Model 1, Random effects (ML), Knapp Hartung, Difference in means TC/HDL-C + LDL-C/HDL-C + Apo B100/Apo A1** | | |  |  |  |  |  |  |  |  |  |  |  |  |  |  |
| --- | --- | --- | --- | --- | --- | --- | --- | --- | --- | --- | --- | --- | --- | --- | --- | --- |
|  | **Current Model** | | **Test of Model (a)** | | | | **Goodness of fit (b)** | | | **Change from prior (c)** | | **Test of change (c)** | | | |  |
|  |  |  |  |  |  |  |  |  |  |  |  |  |  |  |  |  |
| **Covariate** | **Tau²** | **R²** | **F** | **df1** | **df2** | **P-value** | **Q** | **df** | **P-value** | **Tau²** | **R²** | **F** | **df1** | **df2** | **P-value** |  |
| **Intercept** | 0.0144 | 0 |  |  |  |  |  |  |  |  |  |  |  |  |  |  |
| **Intensity VO2max %** | 0.0084 | 0.41 | 1.62 | 1 | 33 | 0.2126 | 41.45 | 33 | 0.1485 | -0.006 | 0.41 | 1.62 | 1 | 33 | 0.2126 | F=1.00, df=4, dfErr=30, p=0.4213 |
| **Intervention Duration (Weeks)** | 0.0069 | 0.52 | 0.87 | 2 | 32 | 0.4286 | 40.92 | 32 | 0.134 | -0.0015 | 0.1 | 0.05 | 1 | 32 | 0.8297 |  |
| **Sessions per week** | 0.0038 | 0.74 | 0.88 | 3 | 31 | 0.4602 | 40.24 | 31 | 0.1238 | -0.0032 | 0.22 | 0.42 | 1 | 31 | 0.5222 |  |
| **Minutes per session** | 0.0023 | 0.84 | 1 | 4 | 30 | 0.4213 | 39.18 | 30 | 0.1217 | -0.0015 | 0.11 | 0.87 | 1 | 30 | 0.3587 |  |

**Table S13b TC/HDL-C + LDL-C/HDL-C + Apo B100/Apo A1 (study variables)**

| **Increments for Model 1, Random effects (ML), Knapp Hartung, Difference in means TC/HDL-C + LDL-C/HDL-C + Ao B100/Apo A1** | | | |  |  |  |  |  |  |  |  |  |  |  |  |  |
| --- | --- | --- | --- | --- | --- | --- | --- | --- | --- | --- | --- | --- | --- | --- | --- | --- |
|  | **Current Model** | | **Test of Model (a)** | | | | **Goodness of fit (b)** | | | **Change from prior (c)(d)** | | **Test of change (c)** | | | |  |
|  |  |  |  |  |  |  |  |  |  |  |  |  |  |  |  |  |
| **Covariate** | **Tau²** | **R²** | **F** | **df1** | **df2** | **P-value** | **Q** | **df** | **P-value** | **Tau²** | **R²** | **F** | **df1** | **df2** | **P-value** |  |
| **Intercept** | 0.0144 | 0 |  |  |  |  |  |  |  |  |  |  |  |  |  |  |
| **Year** | 0.0134 | 0.07 | 0.05 | 1 | 33 | 0.8214 | 43.82 | 33 | 0.0987 | -0.001 | 0.07 | 0.05 | 1 | 33 | 0.8214 | F=0.29, df=4, dfErr=30, p=0.8795 |
| **Total Number of Participants** | 0.0141 | 0.02 | 0.26 | 2 | 32 | 0.774 | 43.69 | 32 | 0.0815 | 0.0007 | -0.05 | 0.47 | 1 | 32 | 0.4969 |  |
| **Number of extracted outcomes** | 0.015 | 0 | 0.17 | 3 | 31 | 0.9145 | 42.67 | 31 | 0.0791 | 0.0009 | -0.02 | 0.01 | 1 | 31 | 0.9143 |  |
| **TESTEX Score** | 0.0174 | 0 | 0.29 | 4 | 30 | 0.8795 | 42.58 | 30 | 0.0638 | 0.0025 | 0 | 0.63 | 1 | 30 | 0.4348 |  |

**Table S14a Apo A1 + Apo A2 + HDL2 + HDL3 mmol/L (intervention variables)**

| **Increments for Model 1, Random effects (ML), Knapp Hartung, Difference in means Apo A1 + Apo A2 + HDL2 + HDL3 mmol/L Intervention variables** | | | | |  |  |  | |  |  |  | |  |  | |  |  |  |  | |  |
| --- | --- | --- | --- | --- | --- | --- | --- | --- | --- | --- | --- | --- | --- | --- | --- | --- | --- | --- | --- | --- | --- |
|  |  |  |  |  |  |  |  | |  |  |  | |  |  | |  |  |  |  | |  |
|  |  |  | |  |  |  |  | |  |  |  | |  |  | |  |  |  |  | |  |
|  | **Current Model** | | **Test of Model (a)** | | | | | **Goodness of fit (b)** | | | | **Change from prior (c)** | | | **Test of change (c)** | | | | |  | |
|  |  |  | |  |  |  |  | |  |  |  | |  |  | |  |  |  |  | |  |
| **Covariate** | **Tau²** | **R²** | | **F** | **df1** | **df2** | **P-value** | | **Q** | **df** | **P-value** | | **Tau²** | **R²** | | **F** | **df1** | **df2** | **P-value** | |  |
| **Intercept** | 0.0002 | 0 | |  |  |  |  | |  |  |  | |  |  | |  |  |  |  | |  |
| **Intensity VO2max %** | 0 | 1.00 | | 1.1 | 1 | 8 | 0.325 | | 6.86 | 8 | 0.5518 | | -0.0002 | 1.00 | | 1.1 | 1 | 8 | 0.325 | | F=1.24, df=4, dfErr=5, p=0.4024 |
| **Intervention Duration (Weeks)** | 0 | 1.00 | | 0.66 | 2 | 7 | 0.5458 | | 6.64 | 7 | 0.4676 | | 0 | 0 | | 0.22 | 1 | 7 | 0.6514 | |  |
| **Sessions per week** | 0 | 1.00 | | 1.65 | 3 | 6 | 0.2759 | | 3.02 | 6 | 0.8062 | | 0 | 0 | | 3.62 | 1 | 6 | 0.1059 | |  |
| **Minutes per session** | 0 | 1.00 | | 1.24 | 4 | 5 | 0.4024 | | 3.02 | 5 | 0.6971 | | 0 | 0 | | 0 | 1 | 5 | 0.9676 | |  |

**Table S14b Apo A1 + Apo A2 + HDL2 + HDL3 mmol/L (study variables)**

| **Increments for Model 1, Random effects (ML), Knapp Hartung, Difference in means**  **Apo A1 + Apo A2 + HDL2 + HDL3 mmol/L**  **Study variables** | | | |  |  |  |  |  |  |  |  |  |  |  |  |  |
| --- | --- | --- | --- | --- | --- | --- | --- | --- | --- | --- | --- | --- | --- | --- | --- | --- |
|  |  |  |  |  |  |  |  |  |  |  |  |  |  |  |  |  |
|  |  |  |  |  |  |  |  |  |  |  |  |  |  |  |  |  |
|  | **Current Model** | | **Test of Model (a)** | | | | **Goodness of fit (b)** | | | **Change from prior (c)** | | **Test of change (c)** | | | |  |
|  |  |  |  |  |  |  |  |  |  |  |  |  |  |  |  |  |
| **Covariate** | **Tau²** | **R²** | **F** | **df1** | **df2** | **P-value** | **Q** | **df** | **P-value** | **Tau²** | **R²** | **F** | **df1** | **df2** | **P-value** |  |
| **Intercept** | 0.0002 | 0 |  |  |  |  |  |  |  |  |  |  |  |  |  |  |
| **Year** | 0 | 1.00 | 1.96 | 1 | 8 | 0.1992 | 6 | 8 | 0.6471 | -0.0002 | 1.00 | 1.96 | 1 | 8 | 0.1992 | F=1.14, df=4, dfErr=5, p=0.4329 |
| **Total Number of Participants** | 0 | 1.00 | 1.34 | 2 | 7 | 0.3225 | 5.29 | 7 | 0.625 | 0 | 0 | 0.71 | 1 | 7 | 0.4262 |  |
| **Number of extracted outcomes** | 0 | 1.00 | 1.11 | 3 | 6 | 0.4153 | 4.63 | 6 | 0.5927 | 0 | 0 | 0.66 | 1 | 6 | 0.447 |  |
| **TESTEX Score** | 0 | 1.00 | 1.14 | 4 | 5 | 0.4329 | 3.39 | 5 | 0.6401 | 0 | 0 | 1.24 | 1 | 5 | 0.317 |  |
